# Prognosis predictive value of the Oxford Acute Severity of Illness Score for sepsis: a retrospective cohort study

**DOI:** 10.7717/peerj.7083

**Published:** 2019-06-10

**Authors:** Qingui Chen, Lishan Zhang, Shanhui Ge, Wanmei He, Mian Zeng

**Affiliations:** Department of Medical Intensive Care Unit, First Affiliated Hospital of Sun Yat-sen University, Guangzhou, Guangdong, China

**Keywords:** Sepsis, Severity of illness index, Critical care outcomes, Mortality

## Abstract

**Background:**

The Oxford Acute Severity of Illness Score (OASIS) has shown fair prognosis predictive value in critically ill patients, but its predictive value has not been assessed in septic patients.

**Objective:**

The aim of this study was to evaluate the performance of the OASIS for the assessment of mortality in septic patients, especially when compared with the Sepsis-related Organ Failure Assessment (SOFA) score.

**Methods:**

A retrospective cohort study was conducted using data from a public database and septic patients were identified using the Sepsis-3 criteria. The primary outcome was hospital mortality. Data were mainly analyzed using multivariable logistic regression and receiver operating characteristic (ROC) curves. Sensitive analyses were performed in patients with an ICD-9-CM code for sepsis and ROC curves analyses were also conducted in septic patients stratified by the Simplified Acute Physiology Score (SAPS) II as subgroup analyses.

**Results:**

A total of 10,305 septic patients were included. The OASIS was found to be significantly associated with hospital mortality (odds ratio 1.07 per one-point increase, 95% confidence interval [1.06–1.08]), while ROC curves analyses showed the discriminatory power of the OASIS for hospital mortality was statistically significantly lower than that of the SOFA score (area under the ROC curve: 0.652 vs 0.682, *p* < 0.001). Results of sensitive analyses were consistent, but the significant difference existed only when the SAPS II was higher than 50 according to results of the subgroup analyses.

**Conclusions:**

The OASIS might serve as an initial predictor of clinical outcomes for septic patients, but one should be circumspect when it is applied to severer patients.

## Introduction

Scoring systems for outcome prediction have been developed in intensive care medicine for a long time, and some of them have been widely used in the intensive care unit (ICU) (Rapsang & Shyam, 2014). For patients with sepsis, the Sepsis-related Organ Failure Assessment (SOFA) score, which was initially designed to evaluate the severity of organ dysfunction in patients who were critically ill from sepsis (Vincent et al., 1998), has been proposed as a tool to facilitate the identification of patients at risk of dying from sepsis (Singer et al., 2016). The SOFA system uses a few measurements of major organ function to calculate a severity score, which contains some laboratory results such as platelets and bilirubin. Other scoring systems which consisted of more complex parameters such as the Simplified Acute Physiology Score II (SAPS II) (Le Gall, Lemeshow & Saulnier, 1993) were also widely used in ICU practice. However, as we know, clinicians usually prefer an instrument that is not laboratory-dependent and is easy to use. The quick SOFA (qSOFA) score, which consists of only three parameters and can be easily measured at the bedside, has also been proposed as a tool to help identify patients with early sepsis outside of the ICU (Seymour et al., 2016), but it was reported to have poor accuracy for predicting 28-day mortality in critically ill septic patients (Hwang et al., 2018). Johnson, Kramer & Clifford (2013) developed a new reduced severity of illness score using machine-learning algorithms, the Oxford Acute Severity of Illness Score (OASIS), which contained 10 parameters without any laboratory tests and had discrimination and calibration equivalent to more complex existing models. Given that the predictive value of the OASIS was only validated in mixed ICU patient populations, its performance in septic patients remains unknown. In this study, we evaluated the association of the OASIS with outcomes of septic patients in ICU, and examined its predictive value mainly by comparison with the SOFA score.

## Methods

### Database

The retrospective cohort study was conducted using data from the Medical Information Mart for Intensive Care III (MIMIC-III) database (v1.4). MIMIC-III is a publicly accessible critical care database which consists of de-identified health-related data about over 40,000 patients stayed in the ICU of Beth Israel Deaconess Medical Center between 2001 and 2012 (Johnson et al., 2016). The access to the database has been approved by the institutional review boards of both Beth Israel Deaconess Medical Center and Massachusetts Institute of Technology Affiliates after completing the CITI (Collaborative Institutional Training Initiative) “Data or Specimens Only Research” course (Qingui Chen (ID: 6533812), Record ID: 24321991).

### Patients

All adult patients (age ≥ 18 years old) in the database with suspected infection and a SOFA score not less than two points within 24 h after ICU admission were screened for purposes of inclusion, but only those of first hospital admission were considered enrolled. Suspected infection was identified using the International Classification of Diseases, Ninth Revision, Clinical Modification (ICD-9-CM) codes (See appendix 1 of the report from Angus et al. (2001)). To exclude records of organ donors or potential typographical errors, patients whose length of ICU stay were less than 24 h or whose length of hospital stay were less than length of ICU stay were excluded. Length of ICU stay was determined only by the first ICU stay. No informed consent was required on the de-identified patients.

### Data extraction

We extracted data from the database using Transact-SQL and codes from the MIMIC Code Repository (https://github.com/MIT-LCP/mimic-code) (Johnson et al., 2018). Variables extracted included age, sex, admission type, ethnicity, mechanical ventilation on first day, renal replacement therapy on first day, comorbidities (Steiner, Elixhauser & Schnaier, 2002), and parameters of the OASIS (Table S1), the SOFA score, and the SAPS II. For parameters of these three scoring systems, only data within 24 h after ICU admission were extracted and missing components for calculation were treated as normal (usually zero). Length of ICU stay, length of hospital stay, and the Elixhauser Comorbidity Index (SID30) (Thompson et al., 2015) were also calculated. Since the database had date of birth of patients who are older than 89 years old shifted to exactly 300 years before admission to obscure their age, we corrected them (age—300+ 89) before analyses.

### Outcomes

The primary outcome was hospital mortality, and the secondary outcomes were ICU mortality and 28-day mortality after ICU admission. The length of ICU stay and length of hospital stay were calculated only for statistical description. ICU mortality and length of ICU stay were determined by the first ICU stay only.

### Statistical analysis

Results were presented as median and 25th–75th percentiles for continuous variables and numbers and percentages for categorical variables unless otherwise stated. Continuous and categorical variables were analyzed using Mann–Whitney *U* and Chi-square tests, respectively, but Fisher’s exact tests were used instead of Chi-squared tests when the expected values in any of the cells of a contingency table are below 10. The associations of OASIS with ICU outcomes were evaluated using multivariable logistic regression or Cox regression analyses. Univariable logistic regression analyses were performed before to examine the determinants of the primary outcome and variables with a *p*-value less than 0.2 were considered to be included in the multivariable analyses. To examine the linearity assumption of logistic regression, the generalized additive model was used to plot the possibly non-linear relation. Schoenfeld residual plots were also employed to examine the proportional hazard assumption of Cox regression. Discriminatory power was determined by comparing the area under the receiver operating characteristic (ROC) curve (AUC) for each score individually using the method of DeLong, DeLong & Clarke-Pearson (1988). Youden’s index was calculated to determine the best threshold of each scoring system and then patients were grouped by the threshold and Kaplan–Meier curves were drawn to visualize their survival. To test the robustness of the results, we performed sensitive analyses in septic patients identified by ICD-9-CM code for sepsis. To examine potential interaction modifiers of the association between OASIS and the primary outcome, logistic regression analyses across different subgroups were performed. For a variable with a *p*-value for interaction less than 0.05, ROC curve analysis was conducted stratified by the variable after professional judgement. Statistical tests were two-sided when the option was available. A *p*-value less than 0.05 was considered to indicate statistical significance, but for multiple comparisons of the AUC of the three scoring systems, Bonferroni correction was conducted by adjusting the critical *p*-value as 0.05/3. Empower (R) (www.empowerstats.com; X&Y solutions, Inc., Boston, MA, USA) and R software, version 3.4.3 (R Core Team, 2016) were used for all statistical analyses.

## Results

### Characteristics and clinical outcomes of the patients

A total of 10,305 patients with sepsis were included finally with a median OASIS of 34 (25th–75th percentiles 28–40). The median age of the subjects was 69 years (25th–75th percentiles 56–80 years) and 5,425 of the 10,305 cases (52.64%) were male. Among them, 5,196 (50.42%) patients required mechanical ventilation on first day and 514 (4.99%) patients required renal replacement therapy on first day. The five most common comorbidities were hypertension (52.95%), fluid and electrolyte disorders (41.49%), congestive heart failure (33.91%), cardiac arrhythmias (33.51%), and deficiency anemia (23.19%). Other characteristics of the patients are presented in Table 1. With regard to their clinical outcomes, the hospital mortality was 12.31% with 1,269 non-survivors and 9,036 survivors, the ICU mortality was 5.84% and the 28-day mortality was 14.91%. The length of ICU stay and hospital stay was 3.48 (25th–75th percentiles 1.98–7.62) and 11.67 (25th–75th percentiles 7.00–19.94) days, respectively.

**Table 1:** Characteristics and comparison between survivors and non-survivors of all patients.

Variable	All patients (*n* = 10,305)	Survivors (*n* = 9,036)	Non-survivors (*n* = 1,269)	*p*
Age (years)	69 (56–80)	68 (55–80)	74 (61–82)	**<0.001**
Male	5,425 (52.64%)	4,742 (52.48%)	683 (53.82%)	0.370
Admission type				**<0.001**
Urgent	334 (3.24%)	290 (3.21%)	44 (3.47%)	
Emergency	9,006 (87.39%)	7,853 (86.91%)	1,153 (90.86%)	
Elective	965 (9.36%)	893 (9.88%)	72 (5.67%)	
Ethnicity				**<0.001**
White	7,482 (72.61%)	6,592 (72.95%)	890 (70.13%)	
Black	847 (8.22%)	760 (8.41%)	87 (6.86%)	
Asian	246 (2.39%)	225 (2.49%)	21 (1.65%)	
Hispanic/Latino	302 (2.93%)	277 (3.07%)	25 (1.97%)	
Other	1,428 (13.86%)	1,182 (13.08%)	246 (19.39%)	
ICU mortality	602 (5.84%)	0 (0.00%)	602 (47.44%)	**<0.001**
28-day mortality	1,536 (14.91%)	413 (4.57%)	1,123 (88.49%)	**<0.001**
Length of ICU stay (days)	3.48 (1.98–7.62)	3.30 (1.94–7.07)	5.00 (2.43–10.73)	**<0.001**
Length of hospital stay (days)	11.67 (7.00–19.94)	11.49 (6.98–19.61)	12.90 (7.40–23.20)	**<0.001**
OASIS on admission	34 (28–40)	33 (28–39)	38 (32–44)	**<0.001**
SAPS II on admission	39 (31–48)	38 (30–46)	50 (40–59)	**<0.001**
SOFA score on admission	5 (3–7)	5 (3–7)	7 (4–10)	**<0.001**
Elixhauser Comorbidity Index (SID30)	12 (4–23)	12 (3–21)	20 (11–29)	**<0.001**
Sepsis (based on ICD-9)	1,845 (17.90%)	1,413 (15.64%)	432 (34.04%)	**<0.001**
Mechanical ventilation on first day	5,196 (50.42%)	4,510 (49.91%)	686 (54.06%)	**0.006**
Renal replacement therapy on first day	514 (4.99%)	423 (4.68%)	91 (7.17%)	**<0.001**
Comorbidities				
Congestive heart failure	3,494 (33.91%)	2,989 (33.08%)	505 (39.80%)	**<0.001**
Cardiac arrhythmias	3,453 (33.51%)	2,948 (32.63%)	505 (39.80%)	**<0.001**
Valvular disease	1,507 (14.62%)	1,331 (14.73%)	176 (13.87%)	0.416
Pulmonary circulation disorder	815 (7.91%)	705 (7.80%)	110 (8.67%)	0.284
Peripheral vascular disorder	1,188 (11.53%)	1,031 (11.41%)	157 (12.37%)	0.315
Hypertension	5,456 (52.95%)	4,868 (53.87%)	588 (46.34%)	**<0.001**
Paralysis	451 (4.38%)	413 (4.57%)	38 (2.99%)	**0.010**
Other neurological disease	1,411 (13.69%)	1,250 (13.83%)	161 (12.69%)	0.266
Chronic pulmonary disease	2,199 (21.34%)	1,941 (21.48%)	258 (20.33%)	0.349
Uncomplicated diabetes	2,142 (20.79%)	1,918 (21.23%)	224 (17.65%)	**0.003**
Complicated diabetes	761 (7.38%)	679 (7.51%)	82 (6.46%)	0.179
Hypothyroidism	1,107 (10.74%)	984 (10.89%)	123 (9.69%)	0.197
Renal failure	1,852 (17.97%)	1,597 (17.67%)	255 (20.09%)	**0.035**
Liver disease	1,023 (9.93%)	791 (8.75%)	232 (18.28%)	**<0.001**
Peptic ulcer	17 (0.16%)	15 (0.17%)	2 (0.16%)	1.000*
AIDS	151 (1.47%)	131 (1.45%)	20 (1.58%)	0.726
Lymphoma	264 (2.56%)	195 (2.16%)	69 (5.44%)	**<0.001**
Metastatic cancer	665 (6.45%)	485 (5.37%)	180 (14.18%)	**<0.001**
Solid tumor	532 (5.16%)	458 (5.07%)	74 (5.83%)	0.250
Rheumatoid arthritis	346 (3.36%)	308 (3.41%)	38 (2.99%)	0.443
Coagulopathy	1,798 (17.45%)	1,417 (15.68%)	381 (30.02%)	**<0.001**
Obesity	595 (5.77%)	549 (6.08%)	46 (3.62%)	**<0.001**
Weight loss	708 (6.87%)	584 (6.46%)	124 (9.77%)	**<0.001**
Fluid and electrolyte disorders	4,276 (41.49%)	3,602 (39.86%)	674 (53.11%)	**<0.001**
Blood loss anemia	276 (2.68%)	242 (2.68%)	34 (2.68%)	0.998
Deficiency anemia	2,390 (23.19%)	2,163 (23.94%)	227 (17.89%)	**<0.001**
Alcohol abuse	782 (7.59%)	688 (7.61%)	94 (7.41%)	0.795
Drug abuse	353 (3.43%)	330 (3.65%)	23 (1.81%)	**<0.001**
Psychoses	429 (4.16%)	404 (4.47%)	25 (1.97%)	**<0.001**
Depression	849 (8.24%)	778 (8.61%)	71 (5.59%)	**<0.001**

**Notes:**

Patients were grouped as survivors and non-survivors determined by hospital mortality status. Data are expressed as median (25th–75th percentiles) or *n* (%) unless otherwise stated. Mann–Whitney *U* and Chi-square (or Fisher’s exact) tests were used to analyze continuous and categorical variables, respectively. Statistical significance (*p* < 0.05) is shown in bold. The asterisk indicated that the Fisher’s exact test was used instead of the Chi-squared test.

ICU, intensive care unit; OASIS, Oxford Acute Severity of Illness Score; SAPS II, Simplified Acute Physiology Score II; SOFA, Sepsis-related Organ Failure Assessment score; ICD, International Classification of Diseases, Ninth Revision; AIDS, Acquired Immune Deficiency Syndrome.

### Association of the OASIS with clinical outcomes of septic patients

**Figure 1:**
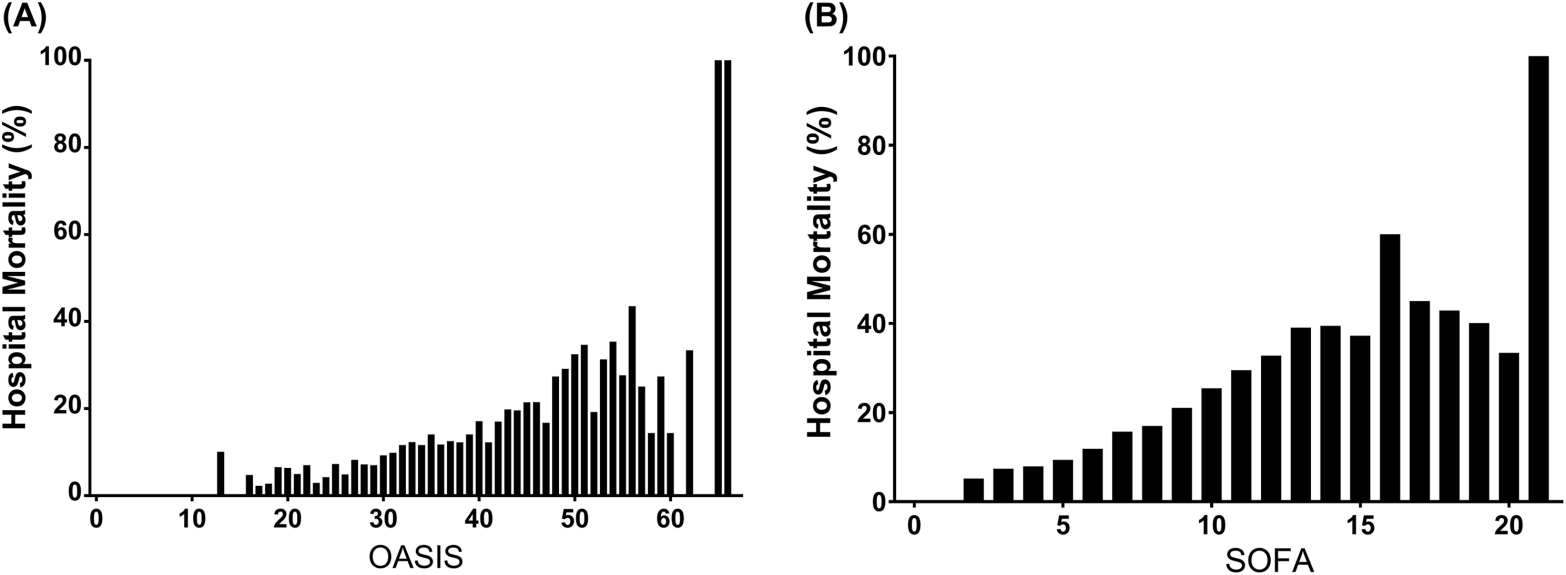
Hospital mortality by different severity scores on ICU admission among patients with sepsis. (A) Hospital mortality by OASIS on ICU admission among patients with sepsis; (B) Hospital mortality by SOFA score on ICU admission among patients with sepsis. Abbreviations: OASIS, Oxford Acute Severity of Illness Score; SOFA, Sepsis-related Organ Failure Assessment score; ICU, intensive care unit.

As shown in Table 1, non-survivors had significantly higher OASIS on ICU admission than survivors (38 vs 33, *p* < 0.001). The distributions of the OASIS and the SOFA score with corresponding hospital mortality are presented in Fig. 1. As each score increased, the hospital mortality of the patients approximately increased accordingly. According to the result of univariable logistic regression analyses for variables associated with hospital mortality which was presented in Table S2, age, admission type, ethnicity, mechanical ventilation on first day, renal replacement therapy on first day, and the Elixhauser Comorbidity Index (SID30) were included in the multivariable regression analyses. Results of curve fitting of the relationship between hospital mortality and several continuous variables were presented in Fig. S1, which indicated that the independent variables were linearly related to the log odds. Schoenfeld residual plots shown in Fig. S2 supported the proportional hazard assumption in Cox models. After adjusting the variables above, the OASIS was significantly associated with hospital mortality (Odds ratio (OR) 1.07 per one-point increase, 95% confidence interval (CI) [1.06–1.08], *p* < 0.0001), ICU mortality (OR 1.07 per one-point increase, 95% CI [1.06–1.08], *p* < 0.001), and 28-day mortality (hazard ratio (HR) 1.06 per one-point increase, 95% CI [1.05–1.06], *p* < 0.001) (Table 2). Results of analyses of the SOFA score and the SAPS II are presented in Tables S3 and S4, respectively.

**Table 2:** Association of OASIS with hospital mortality, ICU mortality, and 28-day mortality.

Outcomes	OR/HR	95% CI	*p*
Hospital mortality			
Non-adjusted	1.07	[1.06–1.08]	**<0.001**
Adjusted	1.07	[1.06–1.08]	**<0.001**
ICU mortality			
Non-adjusted	1.09	[1.08–1.10]	**<0.001**
Adjusted	1.07	[1.06–1.08]	**<0.001**
28-day mortality			
Non-adjusted	1.06	[1.05–1.06]	**<0.001**
Adjusted	1.06	[1.05–1.06]	**<0.001**

**Notes:**

Associations of OASIS with hospital mortality and ICU mortality were analyzed using logistic regression models. Association of OASIS with 28-day mortality was analyzed using Cox regression models. Model was adjusted for age, admission type, ethnicity, mechanical ventilation on first day, renal replacement therapy on first day, and the Elixhauser Comorbidity Index (SID30). Statistical significance (*p* < 0.05) is shown in bold.

OASIS, the Oxford Acute Severity of Illness Score; ICU, intensive care unit; OR, odds ratio; HR, hazard ratio; CI, confidence interval.

### Discriminatory power of the OASIS in septic patients

**Figure 2:**
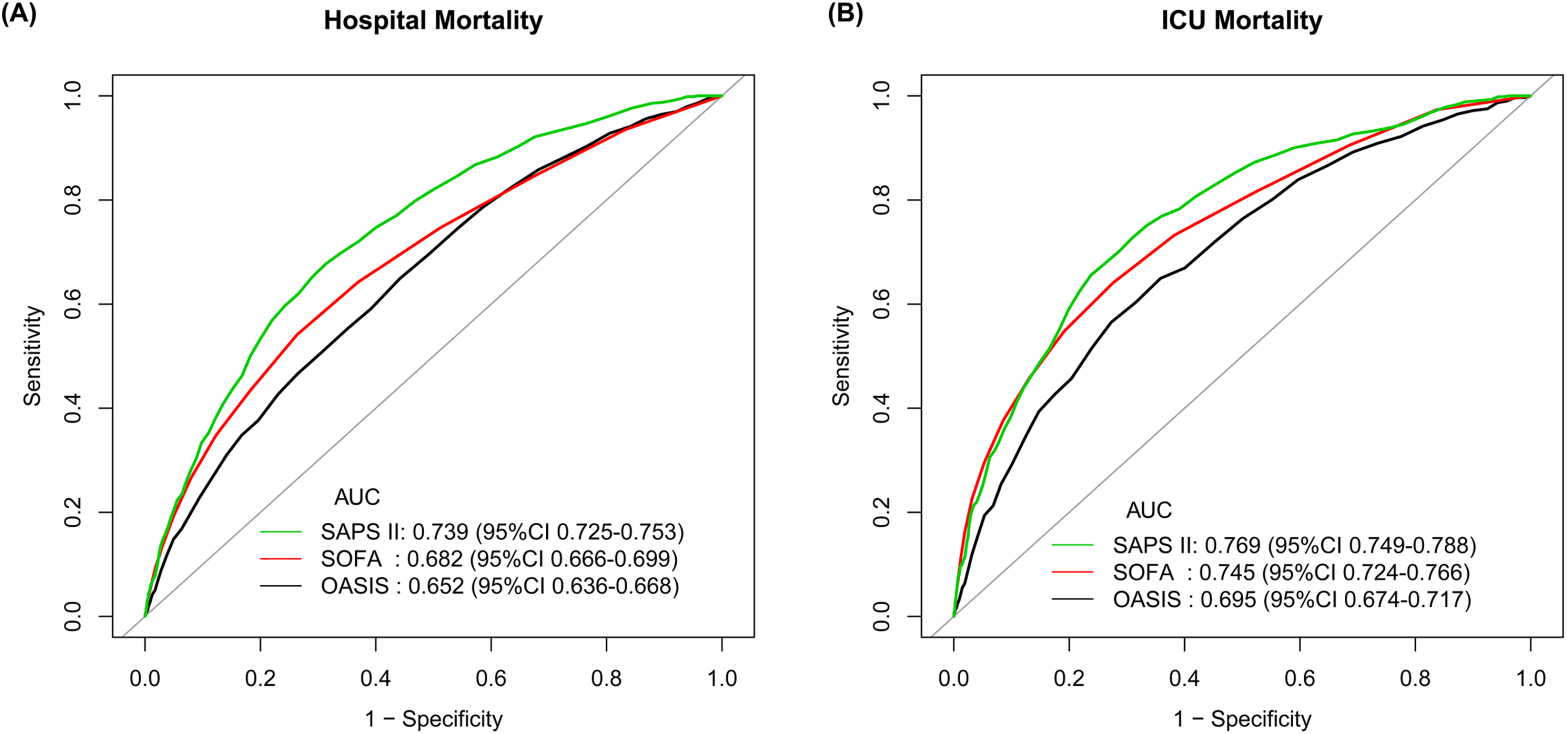
ROC curves assessing discrimination of different severity scores on ICU admission for predicting hospital mortality and ICU mortality. (A) ROC curves for hospital mortality by SAPS II, SOFA score and OASIS; (B) ROC curves for ICU mortality by SAPS II, SOFA score and OASIS. Abbreviations: ROC, receiver operating characteristic; ICU, intensive care unit; SAPS II, Simplified Acute Physiology Score II; SOFA, Sepsis-related Organ Failure Assessment score; OASIS, the Oxford Acute Severity of Illness Score; AUC, area under the ROC curve; CI, confidence interval.

As shown in Fig. 2, the AUC of the OASIS for predicting hospital mortality was 0.652 (95% CI [0.636–0.668]), which was significantly lower than that of the SOFA score (AUC 0.682, 95% CI [0.666–0.699], *p* < 0.001). The best threshold of OASIS was 34.5 with a specificity of 55.80%, a sensitivity of 64.93%, a positive likelihood ratio of 1.47 and a negative likelihood ratio of 0.63. However, the discriminatory power of both scores were all significantly lower than that of the SAPS II (AUC 0.739, 95% CI [0.725–0.753]). Results of comparison of the three scores were similar when they were used to predict ICU mortality (Fig. 2). According to the Kaplan–Meier curves presented in Figs. S3, S4 and S5, patients with higher severity scores had shorter survival time regardless of which scoring system was used.

### Sensitive analyses

**Figure 3:**
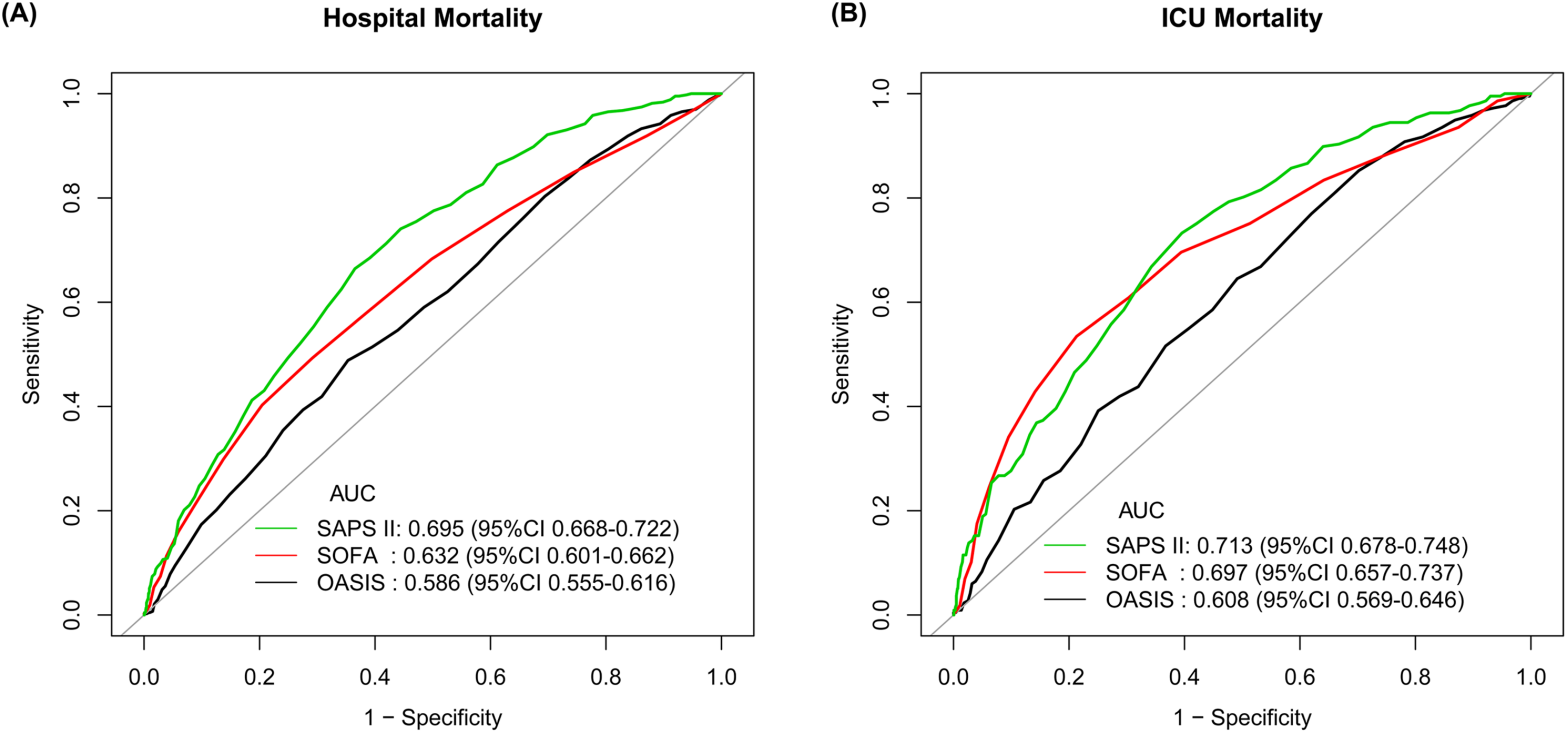
Sensitive analysis of ROC curves assessing discrimination of different severity scores on ICU admission for predicting hospital mortality and ICU mortality. Only patients diagnosed as sepsis according to ICD-9 codes were included into the sensitive analysis. (A) ROC curves for hospital mortality by SAPS II, SOFA score and OASIS; (B) ROC curves for ICU mortality by SAPS II, SOFA score and OASIS. Abbreviations: ROC, receiver operating characteristic; ICU, intensive care unit; SAPS II, Simplified Acute Physiology Score II; SOFA, Sepsis-related Organ Failure Assessment score; OASIS, the Oxford Acute Severity of Illness Score; AUC, area under the ROC curve; CI, confidence interval.

Results of the sensitive analyses were consistent. The OASIS was still significantly associated with hospital mortality (OR = 1.03, 95% CI [1.01–1.05], *p* < 0.001) and 28-day mortality (HR = 1.03, 95% CI [1.01–1.04], *p* < 0.001) when patients with an ICD-9-CM code for sepsis were included only, but no significant association was found between the OASIS and ICU mortality (OR = 1.02, 95% CI [1.00–1.04], *p* = 0.064) (See Table 3). Association of the other two scores with outcomes were presented in Tables S5 and S6, respectively. In the sensitive analyses, the OASIS still had the lowest discriminatory power for predicting hospital mortality (AUC 0.586, 95% CI [0.555–0.616]) and ICU mortality (AUC 0.608, 95% CI [0.569–0.646]) when compared with the SOFA score and the SAPS II (Fig. 3).

**Table 3:** Sensitive analysis of association of OASIS with hospital mortality, ICU mortality, and 28-day mortality.

Outcomes	OR/HR	95% CI	*p*
Hospital mortality			
Non-adjusted	1.04	[1.02–1.05]	**<0.001**
Adjusted	1.03	[1.01–1.05]	**<0.001**
ICU mortality			
Non-adjusted	1.04	[1.03–1.06]	**<0.001**
Adjusted	1.02	[1.00–1.04]	0.064
28-day mortality			
Non-adjusted	1.03	[1.02–1.04]	**<0.001**
Adjusted	1.03	[1.01–1.04]	**<0.001**

**Notes:**

Only patients diagnosed as sepsis according to ICD-9 codes were included into the sensitive analysis. Associations of OASIS with hospital mortality and ICU mortality were analyzed using logistic regression models. Association of OASIS with 28-day mortality was analyzed using Cox regression models. Model was adjusted for age, admission type, ethnicity, mechanical ventilation on first day, renal replacement therapy on first day, and the Elixhauser Comorbidity Index (SID30). Statistical significance (*p* < 0.05) is shown in bold.

OASIS, Oxford Acute Severity of Illness Score; ICU, intensive care unit; OR, odds ratio; HR, hazard ratio; CI, confidence interval.

### Subgroup analyses

**Figure 4:**
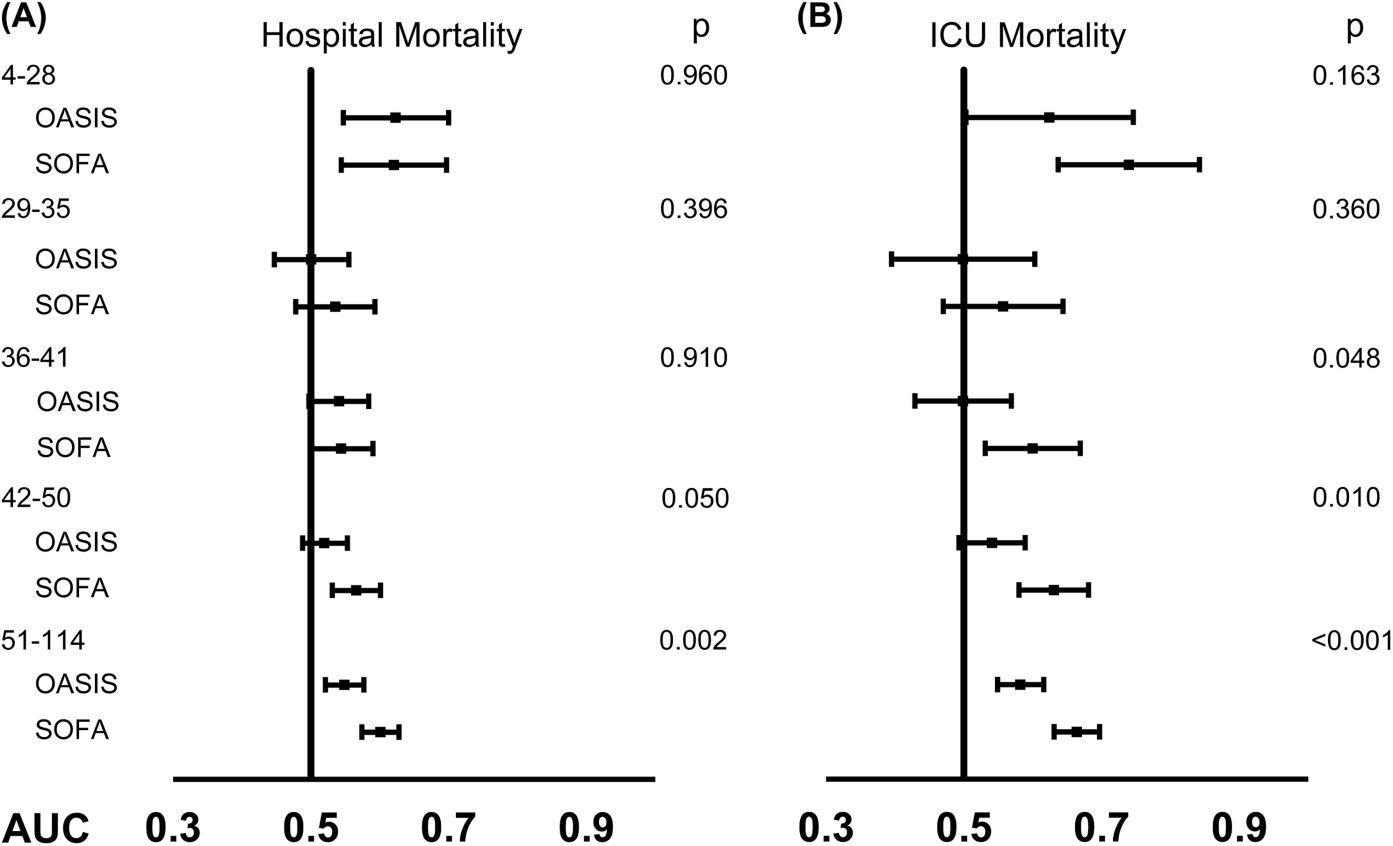
Comparisons of the discriminatory ability of OASIS and SOFA on ICU admission for predicting hospital mortality and ICU mortality stratified by SAPS II. (A) AUCs for hospital mortality by OASIS and SOFA score across different SAPS II categories; (B) AUCs for ICU mortality by OASIS and SOFA score across different SAPS II categories. Abbreviations: OASIS, the Oxford Acute Severity of Illness Score; SOFA, Sepsis-related Organ Failure Assessment score; ICU, intensive care unit; SAPS II, Simplified Acute Physiology Score II; AUC, area under the ROC curve; ROC, receiver operating characteristic.

Associations of the OASIS with hospital mortality were analyzed using logistic regression models across different subgroups, and the result presented in Table S7 found several variables had a *p*-value of interaction less than 0.05. After professional judgement, only the SAPS II was chosen as a potential interaction modifier and examined in the subgroup analysis further. As shown in Fig. 4 and Table S8, there was no statistically significant difference in the discriminatory power of the OASIS and the SOFA score for predicting hospital mortality when a SAPS II was lower than 50. In addition, the SOFA score had significantly higher AUC than that of the OASIS for predicting ICU mortality only when a SAPS II was higher than 35.

## Discussion

In the study, we evaluated the performance of the OASIS for predicting clinical outcomes of patients with sepsis in ICU. Results of our study indicated that the OASIS on ICU admission was significantly associated with short-term clinical outcomes of patients with sepsis, but its discriminatory abilities of hospital mortality and ICU mortality were statistically significantly lower than that of SAPS II. However, an interesting finding was that there was no statistically significant difference between the mortality discriminatory power of the OASIS and that of the SOFA score when the septic patients were not that severe (evaluated by their SAPS II). Even when the patients were more severe, it should be noticed that the absolute AUC difference between the OASIS and the SOFA score was rather small and there was also an overlap between the 95% CIs of the two scores, so it should be cautious when interpreting the clinical significance of the results. Nevertheless, as far as we know, this is the first study that assesses the prognostic predictive value of the OASIS in patients with sepsis in ICU, which might help clinicians to make good use of this newly developed scoring system.

Since the prediction of ICU outcomes might contribute to clinical research and individual patient management (Power & Harrison, 2014), many scoring systems for ICU outcome prediction have been developed and some of them have been routinely used. In terms of sepsis, the most famous scoring system is the SOFA score, an indicator of identifying organ dysfunction and part of the diagnosis criteria of sepsis-3 (Singer et al., 2016). The association of the SOFA score with ICU outcomes has been assessed either in mixed ICU patients or septic patients (Ferreira et al., 2001; Jones, Trzeciak & Kline, 2009; Lie et al., 2018), and it has been reported to be a valuable predictor of ICU outcomes. As shown in Table S3 and Fig. 2, results of our study about the SOFA score were consistent. Unlike the SOFA score, the SAPS II is a widely used disease severity scoring system aimed at mixed ICU patients, and it has been reported to perform better for predicting hospital mortality and 90-day mortality than the SOFA score (Granholm et al., 2016). Similar results were also observed in our study that the SAPS II had the best discriminatory power for hospital mortality and ICU mortality among the three scoring systems analyzed. It is not surprising to find the results, since the SAPS II scoring system has more complicated parameters than the other two systems. However, the complexity of the SAPS II scoring system might be a challenge in daily clinical assessment, which impelled us to investigate the performance of the OASIS.

The OASIS system only consists of 10 easily accessible parameters including length of hospital stay prior ICU admission, age, Glasgow Coma Score (Teasdale & Jennett, 1974), heart rate, mean arterial pressure, respiratory rate, temperature, urine output, ventilation, and elective surgery. In fact, the OASIS system was developed from the popular severity scoring systems APACHE IV (Acute Physiology, Age, and Chronic Health Evaluation VI) with the aim of reducing the number of parameters without losing predictive accuracy (Johnson, Kramer & Clifford, 2013). The goal has been achieved to some extent, however, as far as we know, the discriminatory ability of the OASIS for outcomes of septic patients has not been examined especially when compared with the SOFA score, which has similar numbers of items. Before the study we assumed that the OASIS had poorer prognostic predictive value that that of the SOFA score, for the reason that the OASIS does not contain any laboratory parameters. However, surprisingly, we found that the performance of the OASIS was not different from that of the SOFA score when the septic patients were of mild conditions. Although it is impractical to choose and use the OASIS after the SAPS II of a septic patient has been calculated to determine the severity of the patient, we believe that the OASIS is still a promising tool. Clearly, the simple components of the OASIS make it possible to be calculated automatically, which may provide constructive information especially when few laboratory examinations have been done. In addition, in our previous study (Chen et al., 2018) we have found the OASIS might serve as a supplement to the qSOFA score and help to identify more septic patients. Therefore, we reason that perhaps it might also serve as a supplement to the SOFA score to predict outcomes of septic patients. However, further researches are needed to explore the possibilities.

Another issue needed to be discussed is the criteria we used to identify sepsis. In the study, we initially identified septic patients from the MIMIC-3 database using Sepsis-3 criteria. However, this method assumed a causation from infection to organ dysfunction (the SOFA score ≥2) while in a lot of cases there might be only associations, which might lead to an overestimation of case numbers and an underestimation of mortality (Fleischmann-Struzek et al., 2018). Thus, we then performed sensitive analyses using the ICD-9-CM codes to identify sepsis. Results of the sensitive analyses were consistent, indicating the robustness of our conclusions. However, it should be noticed that the two criteria might represent two distinct populations of sepsis, since the Sepsis-3 criteria was developed in 2016. In our study, the patients identified by the ICD-9-CM codes had higher hospital mortality rate (23.41%) with a median SAPS II of 49.5 and a median SOFA score of 6, so it is not strange to find a lower AUC of the OASIS for hospital mortality in the sensitive analyses since they were more severe.

Several limitations of the study should be noticed. First of all, given the observational nature of our study, selection bias was inevitable although we adjusted a few potential confounders. Second, only the discriminatory power of the OASIS was evaluated, we did not assess its calibration. Third, several variables were not considered. The actual admission years of the patients in the database had been shifted to protect patient confidentiality, so we did not include it in the regression models. Since the database included patients from 2001 to 2012, there might be some differences in outcomes of the patients treated in the early 2000s and later, but we did not take therapy as a covariate into consideration. Last but not least, since all the data in the study was treated as a single dataset, the performance of the OASIS was inherently optimistic.

## Conclusions

To sum up, the retrospective observational study validated significant associations of the OASIS on ICU admission with short-term outcomes of septic patients, and found that although the discriminatory power of the OASIS for hospital mortality was statistically significantly lower than that of the SOFA score, the significant difference existed only when a SAPS II was higher than 50, which suggesting that the OASIS might serve as an initial predictor of clinical outcomes for septic patients, but one should be circumspect when it is applied to more severe patients.

## Supplemental Information

10.7717/peerj.7083/supp-1 Supplemental Information 1 Parameters of the Oxford acute severity of illness score.

10.7717/peerj.7083/supp-2 Supplemental Information 2 Univariable logistic regression for variables associated with hospital mortality. Abbreviations: OR, odds ratio; CI, confidence interval; OASIS, Oxford acute severity of illness score; SAPS II, simplified acute physiology score II; SOFA, Sepsis-related organ failure assessment score.

10.7717/peerj.7083/supp-3 Supplemental Information 3 Association of SOFA score with hospital mortality, ICU mortality, and 28-day mortality. Notes: Associations of SOFA score with hospital mortality and ICU mortality were analyzed using logistic regression models. Association of SOFA score with 28-day mortality was analyzed using Cox regression models. Model was adjusted for age, admission type, ethnicity, mechanical ventilation on first day, renal replacement therapy on first day, and the Elixhauser Comorbidity Index (SID30). Abbreviations: SOFA, Sepsis-related organ failure assessment score; ICU, intensive care unit; OR, odds ratio; HR, hazard ratio; CI, confidence interval.

10.7717/peerj.7083/supp-4 Supplemental Information 4 Association of SAPS II with hospital mortality, ICU mortality, and 28-day mortality. Notes: Associations of SAPS II with hospital mortality and ICU mortality were analyzed using logistic regression models. Association of SAPS II score with 28-day mortality was analyzed using Cox regression models. Model was adjusted for age, admission type, ethnicity, mechanical ventilation on first day, renal replacement therapy on first day, and the Elixhauser Comorbidity Index (SID30). Abbreviations: SAPS II, simplified acute physiology score II; ICU, intensive care unit; OR, odds ratio; HR, hazard ratio; CI, confidence interval.

10.7717/peerj.7083/supp-5 Supplemental Information 5 Sensitive analysis of association of SOFA score with hospital mortality, ICU mortality, and 28-day mortality. Notes: Only patients diagnosed as sepsis according to ICD-9 codes were included into the sensitive analysis. Associations of SOAF score with hospital mortality and ICU mortality were analyzed using logistic regression models. Association of SOFA score with 28-day mortality was analyzed using Cox regression models. Model was adjusted for age, admission type, ethnicity, mechanical ventilation on first day, renal replacement therapy on first day, and the Elixhauser Comorbidity Index (SID30). Abbreviations: SOFA, Sepsis-related organ failure assessment score; ICU, intensive care unit; OR, odds ratio; HR, hazard ratio; CI, confidence interval.

10.7717/peerj.7083/supp-6 Supplemental Information 6 Sensitive analysis of association of SAPS II with hospital mortality, ICU mortality, and 28-day mortality. Notes: Only patients diagnosed as sepsis according to ICD-9 codes were included into the sensitive analysis. Associations of SAPS II with hospital mortality and ICU mortality were analyzed using logistic regression models. Association of SAPS II with 28-day mortality was analyzed using Cox regression models. Model was adjusted for age, admission type, ethnicity, mechanical ventilation on first day, renal replacement therapy on first day, and the Elixhauser Comorbidity Index (SID30). Abbreviations: SAPS II, simplified acute physiology score II; ICU, intensive care unit; OR, odds ratio; HR, hazard ratio; CI, confidence interval.

10.7717/peerj.7083/supp-7 Supplemental Information 7 Potential Interaction modifiers of the association between OASIS and hospital mortality. Notes: Associations of OASIS with hospital mortality were analyzed using logistic regression models across different subgroups to examine potential interaction modifiers. Categories of continuous variables were presented as minimum to maximum. Abbreviations: OASIS, Oxford acute severity of illness score; OR, odds ratio; CI, confidence interval; SAPS II, simplified acute physiology score II; SOFA, Sepsis-related organ failure assessment score; ICD, International Classification of Diseases, Ninth Revision; AIDS, acquired immune deficiency syndrome.

10.7717/peerj.7083/supp-8 Supplemental Information 8 ROC analyses of OASIS and SOFA on ICU admission for predicting hospital mortality and ICU mortality stratified by SAPS II. Abbreviations: ROC, receiver operating characteristic; OASIS, Oxford acute severity of illness score; SOFA, Sepsis-related organ failure assessment score; ICU, intensive care unit; SAPS II, simplified acute physiology score II; AUC, area under the ROC curve; CI, confidence interval.

10.7717/peerj.7083/supp-9 Supplemental Information 9 Curve fitting of the relationship between hospital mortality and several continuous variables. Abbreviations: OASIS, Oxford acute severity of illness score.

10.7717/peerj.7083/supp-10 Supplemental Information 10 Schoenfeld residual plots for testing the proportional hazard assumption in Cox models. Abbreviations: OASIS, Oxford acute severity of illness score.

10.7717/peerj.7083/supp-11 Supplemental Information 11 Kaplan-Meier curves for 28-day mortality according to levels of OASIS on ICU admission. Abbreviations: OASIS, Oxford acute severity of illness score; ICU, intensive care unit.

10.7717/peerj.7083/supp-12 Supplemental Information 12 Kaplan-Meier curves for 28-day mortality according to levels of SOFA on ICU admission. Abbreviations: SOFA, Sepsis-related organ failure assessment score; ICU, intensive care unit.

10.7717/peerj.7083/supp-13 Supplemental Information 13 Kaplan-Meier curves for 28-day mortality according to levels of SAPS II on ICU admission. Abbreviations: SAPS II, simplified acute physiology score II; ICU, intensive care unit.

10.7717/peerj.7083/supp-14 Supplemental Information 14 Raw codes. The main codes used for extracting data are presented. More basic codes are available at https://github.com/MIT-LCP/mimic-code.

## Additional Information and Declarations

### Data Availability

The following information was supplied regarding data availability: The codes we created are available in the Supplemental File to help readers to replicate the analyses. More codes can be found at https://github.com/MIT-LCP/mimic-code. The raw data itself is from a third-party dataset available from MIMIC-III, a freely accessible critical care database. Reproduction of their data is not permitted according to the Data Use Agreement of the database but access can be requested here: https://mimic.physionet.org/gettingstarted/access.
