# Supplementary material for: Prognosis predictive value of the Oxford Acute Severity of Illness Score for sepsis: a retrospective cohort study
Source: PeerJ. 2019 Jun 10;7:e7083. doi: 10.7717/peerj.7083 (PMC6563807; doi:10.7717/peerj.7083)
Supplement: Supplemental Information 1 [file peerj-07-7083-s001.docx]

| Variables | Score |
| --- | --- |
| Pre-ICU length of stay (hours) |  |
| <0.17 | 5 |
| 0.17-4.94 | 3 |
| 4.95-24.00 | 0 |
| 24.01-311.80 | 2 |
| >311.80 | 1 |
| Age (years) |  |
| <24 | 0 |
| 24-53 | 3 |
| 54-77 | 6 |
| 78-89 | 9 |
| ≥90 | 7 |
| Glasgow Coma Score |  |
| 3-7 | 10 |
| 8-13 | 4 |
| 14 | 3 |
| 15 | 0 |
| Heart rate (min^-1^) |  |
| <33 | 4 |
| 33-88 | 0 |
| 89-106 | 1 |
| 107-125 | 3 |
| >125 | 6 |
| Mean arterial pressure (mmHg) |  |
| <20.65 | 4 |
| 20.65-50.99 | 3 |
| 51-61.32 | 2 |
| 61.33-143.44 | 0 |
| >143.44 | 3 |
| Respiratory rate (min^-1^) |  |
| <6 | 10 |
| 6-12 | 1 |
| 13-22 | 0 |
| 23-30 | 1 |
| 31-44 | 6 |
| >44 | 9 |
| Temperature (℃) |  |
| <33.22 | 3 |
| 33.22-35.93 | 4 |
| 35.94-36.39 | 2 |
| 36.40-36.88 | 0 |
| 36.89-39.88 | 2 |
| >39.88 | 6 |
| Urine output (ml/day) |  |
| <671 | 10 |
| 671-1426.99 | 5 |
| 1427-2543.99 | 1 |
| 2544-6896 | 0 |
| >6896 | 8 |
| Ventilated |  |
| No | 0 |
| Yes | 9 |
| Elective Surgery |  |
| Yes | 0 |
| No | 6 |
