# Supplementary material for: Prognosis predictive value of the Oxford Acute Severity of Illness Score for sepsis: a retrospective cohort study
Source: PeerJ. 2019 Jun 10;7:e7083. doi: 10.7717/peerj.7083 (PMC6563807; doi:10.7717/peerj.7083)
Supplement: Supplemental Information 2 — Abbreviations: OR, odds ratio; CI, confidence interval; OASIS, Oxford acute severity of illness score; SAPS II, simplified acute physiology score II; SOFA, Sepsis-related organ failure assessment score. [file peerj-07-7083-s002.docx]

| Variables | OR | 95% CI | p |
| --- | --- | --- | --- |
| Age (years) | 1.02 | 1.01-1.02 | **<0.001** |
| Sex |  |  |  |
| Male | 1.0 |  |  |
| Female | 0.95 | 0.84-1.07 | 0.370 |
| Admission type |  |  |  |
| Urgent | 1.0 |  |  |
| Emergency | 0.97 | 0.70-1.34 | 0.842 |
| Elective | 0.53 | 0.36-0.79 | **0.002** |
| Ethnicity |  |  |  |
| White | 1.0 |  |  |
| Black | 0.85 | 0.67-1.07 | 0.164 |
| Asian | 0.69 | 0.44-1.09 | 0.110 |
| Hispanic/Latino | 0.67 | 0.44-1.01 | 0.057 |
| Other | 1.54 | 1.32-1.80 | **<0.001** |
| OASIS on admission | 1.07 | 1.06-1.08 | **<0.001** |
| SAPS II on admission | 1.06 | 1.06-1.07 | **<0.001** |
| SOFA on admission | 1.23 | 1.21-1.25 | **<0.001** |
| Elixhauser Comorbidity Index (SID30) | 1.04 | 1.04-1.05 | **<0.001** |
| Mechanical ventilation on first day |  |  |  |
| No | 1.0 |  |  |
| Yes | 1.18 | 1.05-1.33 | **0.006** |
| Renal replacement therapy on first day |  |  |  |
| No | 1.0 |  |  |
| Yes | 1.57 | 1.24-1.99 | **<0.001** |
