# Supplementary material for: Prognosis predictive value of the Oxford Acute Severity of Illness Score for sepsis: a retrospective cohort study
Source: PeerJ. 2019 Jun 10;7:e7083. doi: 10.7717/peerj.7083 (PMC6563807; doi:10.7717/peerj.7083)
Supplement: Supplemental Information 4 — Notes: Associations of SAPS II with hospital mortality and ICU mortality were analyzed using logistic regression models. Association of SAPS II score with 28-day mortality was analyzed using Cox regression models. Model was adjusted for age, admission type, ethnicity, mechanical ventilation on first day, renal replacement therapy on first day, and the Elixhauser Comorbidity Index (SID30). Abbreviations: SAPS II, simplified acute physiology score II; ICU, intensive care unit; OR, odds ratio; HR, hazard ratio; CI, confidence interval. [file peerj-07-7083-s004.docx]

| Outcomes | OR/HR | 95% CI | p |
| --- | --- | --- | --- |
| Hospital mortality |  |  |  |
| Non-adjusted | 1.06 | 1.06-1.07 | **<0.001** |
| Adjusted | 1.06 | 1.05-1.07 | **<0.001** |
| ICU mortality |  |  |  |
| Non-adjusted | 1.07 | 1.06-1.08 | **<0.001** |
| Adjusted | 1.06 | 1.06-1.07 | **<0.001** |
| 28-day mortality |  |  |  |
| Non-adjusted | 1.05 | 1.05-1.05 | **<0.001** |
| Adjusted | 1.05 | 1.05-1.05 | **<0.001** |
