# Supplementary material for: Prognosis predictive value of the Oxford Acute Severity of Illness Score for sepsis: a retrospective cohort study
Source: PeerJ. 2019 Jun 10;7:e7083. doi: 10.7717/peerj.7083 (PMC6563807; doi:10.7717/peerj.7083)
Supplement: Supplemental Information 7 — Notes: Associations of OASIS with hospital mortality were analyzed using logistic regression models across different subgroups to examine potential interaction modifiers. Categories of continuous variables were presented as minimum to maximum. Abbreviations: OASIS, Oxford acute severity of illness score; OR, odds ratio; CI, confidence interval; SAPS II, simplified acute physiology score II; SOFA, Sepsis-related organ failure assessment score; ICD, International Classification of Diseases, Ninth Revision; AIDS, acquired immune deficiency syndrome. [file peerj-07-7083-s007.docx]

| Modifiers | OR | 95% CI | p | p interaction |
| --- | --- | --- | --- | --- |
| Age categories |  |  |  | 0.740 |
| Q1 (18-60) | 1.06 | 1.05-1.08 | **<0.001** |  |
| Q2 (61-77) | 1.07 | 1.05-1.08 | **<0.001** |  |
| Q3 (78-99) | 1.07 | 1.05-1.08 | **<0.001** |  |
| Total | 1.07 | 1.06-1.07 | **<0.001** |  |
| Sex |  |  |  | 0.256 |
| Male | 1.07 | 1.06-1.08 | **<0.001** |  |
| Female | 1.06 | 1.05-1.08 | **<0.001** |  |
| Total | 1.07 | 1.06-1.08 | **<0.001** |  |
| Admission type |  |  |  | 0.234 |
| Urgent | 1.05 | 1.01-1.10 | **0.009** |  |
| Emergency | 1.07 | 1.06-1.07 | **<0.001** |  |
| Elective | 1.09 | 1.06-1.13 | **<0.001** |  |
| Total | 1.07 | 1.06-1.07 | **<0.001** |  |
| Ethnicity |  |  |  | 0.681 |
| White | 1.07 | 1.06-1.08 | **<0.001** |  |
| Black | 1.07 | 1.05-1.10 | **<0.001** |  |
| Asian | 1.08 | 1.02-1.14 | **0.008** |  |
| Hispanic/Latino | 1.10 | 1.05-1.16 | **<0.001** |  |
| Other | 1.06 | 1.04-1.08 | **<0.001** |  |
| Total | 1.07 | 1.06-1.08 | **<0.001** |  |
| SAPS II categories |  |  |  | **<0.001** |
| Q1 (4-28) | 1.07 | 1.03-1.12 | **<0.001** |  |
| Q2 (29-35) | 1.00 | 0.97-1.03 | 0.838 |  |
| Q3 (36-41) | 0.97 | 0.95-1.00 | **0.030** |  |
| Q4 (42-50) | 1.01 | 1.00-1.03 | 0.144 |  |
| Q5 (51-114) | 1.02 | 1.01-1.04 | **<0.001** |  |
| Total | 1.01 | 1.00-1.02 | **0.004** |  |
| SOFA categories |  |  |  | 0.419 |
| Q1 (2-3) | 1.06 | 1.04-1.08 | **<0.001** |  |
| Q2 (4-5) | 1.05 | 1.03-1.07 | **<0.001** |  |
| Q3 (6-21) | 1.05 | 1.04-1.06 | **<0.001** |  |
| Total | 1.05 | 1.04-1.06 | **<0.001** |  |
| Elixhauser Comorbidity Index (SID30) categories |  |  |  | **0.003** |
| Q1 (-22-1) | 1.09 | 1.06-1.13 | **<0.001** |  |
| Q2 (2-10) | 1.08 | 1.06-1.10 | **<0.001** |  |
| Q3 (11-16) | 1.06 | 1.04-1.08 | **<0.001** |  |
| Q4 (17-24) | 1.06 | 1.05-1.08 | **<0.001** |  |
| Q5 (25-74) | 1.04 | 1.03-1.06 | **<0.001** |  |
| Total | 1.06 | 1.05-1.07 | **<0.001** |  |
| Sepsis (based on ICD-9) |  |  |  | **<0.001** |
| No | 1.07 | 1.06-1.08 | **<0.001** |  |
| Yes | 1.04 | 1.02-1.05 | **<0.001** |  |
| Total | 1.06 | 1.05-1.07 | **<0.001** |  |
| Mechanical ventilation on first day |  |  |  | 0.611 |
| No | 1.09 | 1.07-1.10 | **<0.001** |  |
| Yes | 1.08 | 1.07-1.09 | **<0.001** |  |
| Total | 1.08 | 1.07-1.09 | **<0.001** |  |
| Renal replacement therapy on first day |  |  |  | 0.165 |
| No | 1.07 | 1.06-1.08 | **<0.001** |  |
| Yes | 1.05 | 1.02-1.08 | **<0.001** |  |
| Total | 1.07 | 1.06-1.08 | **<0.001** |  |
| Congestive heart failure |  |  |  | **0.020** |
| No | 1.07 | 1.06-1.08 | **<0.001** |  |
| Yes | 1.06 | 1.04-1.07 | **<0.001** |  |
| Total | 1.07 | 1.06-1.08 | **<0.001** |  |
| Cardiac arrhythmias |  |  |  | **0.049** |
| No | 1.07 | 1.06-1.08 | **<0.001** |  |
| Yes | 1.06 | 1.04-1.07 | **<0.001** |  |
| Total | 1.07 | 1.06-1.07 | **<0.001** |  |
| Valvular disease |  |  |  | 0.518 |
| No | 1.07 | 1.06-1.08 | **<0.001** |  |
| Yes | 1.06 | 1.04-1.08 | **<0.001** |  |
| Total | 1.07 | 1.06-1.08 | **<0.001** |  |
| Pulmonary circulation disorder |  |  |  | 0.610 |
| No | 1.07 | 1.06-1.08 | **<0.001** |  |
| Yes | 1.06 | 1.04-1.09 | **<0.001** |  |
| Total | 1.07 | 1.06-1.08 | **<0.001** |  |
| Peripheral vascular disorder |  |  |  | 0.081 |
| No | 1.07 | 1.06-1.08 | **<0.001** |  |
| Yes | 1.05 | 1.03-1.07 | **<0.001** |  |
| Total | 1.07 | 1.06-1.08 | **<0.001** |  |
| Hypertension |  |  |  | 0.781 |
| No | 1.07 | 1.06-1.08 | **<0.001** |  |
| Yes | 1.07 | 1.06-1.08 | **<0.001** |  |
| Total | 1.07 | 1.06-1.08 | **<0.001** |  |
| Paralysis |  |  |  | 0.395 |
| No | 1.07 | 1.06-1.08 | **<0.001** |  |
| Yes | 1.05 | 1.01-1.09 | **0.026** |  |
| Total | 1.07 | 1.06-1.08 | **<0.001** |  |
| Other neurological disease |  |  |  | 0.426 |
| No | 1.07 | 1.06-1.08 | **<0.001** |  |
| Yes | 1.06 | 1.04-1.08 | **<0.001** |  |
| Total | 1.07 | 1.06-1.08 | **<0.001** |  |
| Chronic pulmonary disease |  |  |  | 0.542 |
| No | 1.07 | 1.06-1.08 | **<0.001** |  |
| Yes | 1.06 | 1.05-1.08 | **<0.001** |  |
| Total | 1.07 | 1.06-1.08 | **<0.001** |  |
| Uncomplicated diabetes |  |  |  | 0.907 |
| No | 1.07 | 1.06-1.08 | **<0.001** |  |
| Yes | 1.07 | 1.05-1.09 | **<0.001** |  |
| Total | 1.07 | 1.06-1.08 | **<0.001** |  |
| Complicated diabetes |  |  |  | 0.450 |
| No | 1.07 | 1.06-1.08 | **<0.001** |  |
| Yes | 1.08 | 1.05-1.11 | **<0.001** |  |
| Total | 1.07 | 1.06-1.08 | **<0.001** |  |
| Hypothyroidism |  |  |  | 0.386 |
| No | 1.07 | 1.06-1.08 | **<0.001** |  |
| Yes | 1.06 | 1.03-1.08 | **<0.001** |  |
| Total | 1.07 | 1.06-1.08 | **<0.001** |  |
| Renal failure |  |  |  | 0.310 |
| No | 1.07 | 1.06-1.08 | **<0.001** |  |
| Yes | 1.06 | 1.04-1.08 | **<0.001** |  |
| Total | 1.07 | 1.06-1.08 | **<0.001** |  |
| Liver disease |  |  |  | 0.985 |
| No | 1.07 | 1.06-1.08 | **<0.001** |  |
| Yes | 1.07 | 1.05-1.09 | **<0.001** |  |
| Total | 1.07 | 1.06-1.08 | **<0.001** |  |
| AIDS |  |  |  | 0.071 |
| No | 1.07 | 1.06-1.08 | **<0.001** |  |
| Yes | 1.02 | 0.97-1.07 | 0.494 |  |
| Total | 1.07 | 1.06-1.08 | **<0.001** |  |
| Lymphoma |  |  |  | 0.768 |
| No | 1.07 | 1.06-1.08 | **<0.001** |  |
| Yes | 1.07 | 1.04-1.11 | **<0.001** |  |
| Total | 1.07 | 1.06-1.08 | **<0.001** |  |
| Metastatic cancer |  |  |  | 0.190 |
| No | 1.07 | 1.06-1.08 | **<0.001** |  |
| Yes | 1.06 | 1.03-1.08 | **<0.001** |  |
| Total | 1.07 | 1.06-1.08 | **<0.001** |  |
| Solid tumor |  |  |  | 0.487 |
| No | 1.07 | 1.06-1.08 | **<0.001** |  |
| Yes | 1.08 | 1.05-1.11 | **<0.001** |  |
| Total | 1.07 | 1.06-1.08 | **<0.001** |  |
| Rheumatoid arthritis |  |  |  | 0.922 |
| No | 1.07 | 1.06-1.08 | **<0.001** |  |
| Yes | 1.07 | 1.02-1.11 | **0.002** |  |
| Total | 1.07 | 1.06-1.08 | **<0.001** |  |
| Coagulopathy |  |  |  | **0.004** |
| No | 1.07 | 1.07-1.08 | **<0.001** |  |
| Yes | 1.05 | 1.03-1.06 | **<0.001** |  |
| Total | 1.07 | 1.06-1.07 | **<0.001** |  |
| Obesity |  |  |  | 0.769 |
| No | 1.07 | 1.06-1.08 | **<0.001** |  |
| Yes | 1.06 | 1.03-1.10 | **<0.001** |  |
| Total | 1.07 | 1.06-1.08 | **<0.001** |  |
| Weight loss |  |  |  | **0.045** |
| No | 1.07 | 1.06-1.08 | **<0.001** |  |
| Yes | 1.04 | 1.02-1.07 | **<0.001** |  |
| Total | 1.07 | 1.06-1.08 | **<0.001** |  |
| Fluid and electrolyte disorders |  |  |  | 0.088 |
| No | 1.07 | 1.06-1.08 | **<0.001** |  |
| Yes | 1.06 | 1.05-1.07 | **<0.001** |  |
| Total | 1.07 | 1.06-1.07 | **<0.001** |  |
| Blood loss anemia |  |  |  | 0.462 |
| No | 1.07 | 1.06-1.08 | **<0.001** |  |
| Yes | 1.05 | 1.01-1.10 | **0.021** |  |
| Total | 1.07 | 1.06-1.08 | **<0.001** |  |
| Deficiency anemia |  |  |  | 0.140 |
| No | 1.07 | 1.06-1.08 | **<0.001** |  |
| Yes | 1.06 | 1.04-1.07 | **<0.001** |  |
| Total | 1.07 | 1.06-1.08 | **<0.001** |  |
| Alcohol abuse |  |  |  | 0.371 |
| No | 1.07 | 1.06-1.08 | **<0.001** |  |
| Yes | 1.06 | 1.03-1.08 | **<0.001** |  |
| Total | 1.07 | 1.06-1.08 | **<0.001** |  |
| Drug abuse |  |  |  | 0.556 |
| No | 1.07 | 1.06-1.08 | **<0.001** |  |
| Yes | 1.05 | 0.99-1.11 | 0.094 |  |
| Total | 1.07 | 1.06-1.08 | **<0.001** |  |
| Psychoses |  |  |  | 0.508 |
| No | 1.07 | 1.06-1.08 | **<0.001** |  |
| Yes | 1.05 | 1.00-1.11 | 0.074 |  |
| Total | 1.07 | 1.06-1.08 | **<0.001** |  |
| Depression |  |  |  | 0.061 |
| No | 1.07 | 1.06-1.07 | **<0.001** |  |
| Yes | 1.10 | 1.07-1.13 | **<0.001** |  |
| Total | 1.07 | 1.06-1.08 | **<0.001** |  |
