# Supplementary material for: Prognosis predictive value of the Oxford Acute Severity of Illness Score for sepsis: a retrospective cohort study
Source: PeerJ. 2019 Jun 10;7:e7083. doi: 10.7717/peerj.7083 (PMC6563807; doi:10.7717/peerj.7083)
Supplement: Supplemental Information 8 — Abbreviations: ROC, receiver operating characteristic; OASIS, Oxford acute severity of illness score; SOFA, Sepsis-related organ failure assessment score; ICU, intensive care unit; SAPS II, simplified acute physiology score II; AUC, area under the ROC curve; CI, confidence interval. [file peerj-07-7083-s008.docx]

|  | Hospital Mortality | | | ICU Mortality | | |
| --- | --- | --- | --- | --- | --- | --- |
| SAPS II | AUC | 95% CI | p | AUC | 95% CI | p |
| 4-28 |  |  | 0.960 |  |  | 0.163 |
| OASIS | 0.623 | 0.547-0.700 |  | 0.624 | 0.503-0.746 |  |
| SOFA | 0.621 | 0.544-0.697 |  | 0.740 | 0.637-0.842 |  |
| 29-35 |  |  | 0.396 |  |  | 0.360 |
| OASIS | 0.501 | 0.447-0.555 |  | 0.499 | 0.395-0.603 |  |
| SOFA | 0.536 | 0.478-0.593 |  | 0.557 | 0.470-0.644 |  |
| 36-41 |  |  | 0.910 |  |  | **0.048** |
| OASIS | 0.541 | 0.497-0.584 |  | 0.499 | 0.429-0.569 |  |
| SOFA | 0.544 | 0.499-0.590 |  | 0.600 | 0.531-0.669 |  |
| 42-50 |  |  | 0.050 |  |  | **0.010** |
| OASIS | 0.520 | 0.488-0.553 |  | 0.541 | 0.493-0.589 |  |
| SOFA | 0.566 | 0.531-0.601 |  | 0.631 | 0.580-0.681 |  |
| 51-114 |  |  | **0.002** |  |  | **<0.001** |
| OASIS | 0.549 | 0.521-0.577 |  | 0.582 | 0.549-0.616 |  |
| SOFA | 0.601 | 0.574-0.628 |  | 0.664 | 0.631-0.697 |  |
