# Supplementary figures and images for: Prognosis predictive value of the Oxford Acute Severity of Illness Score for sepsis: a retrospective cohort study
Source: PeerJ. 2019 Jun 10;7:e7083. doi: 10.7717/peerj.7083 (PMC6563807; doi:10.7717/peerj.7083)

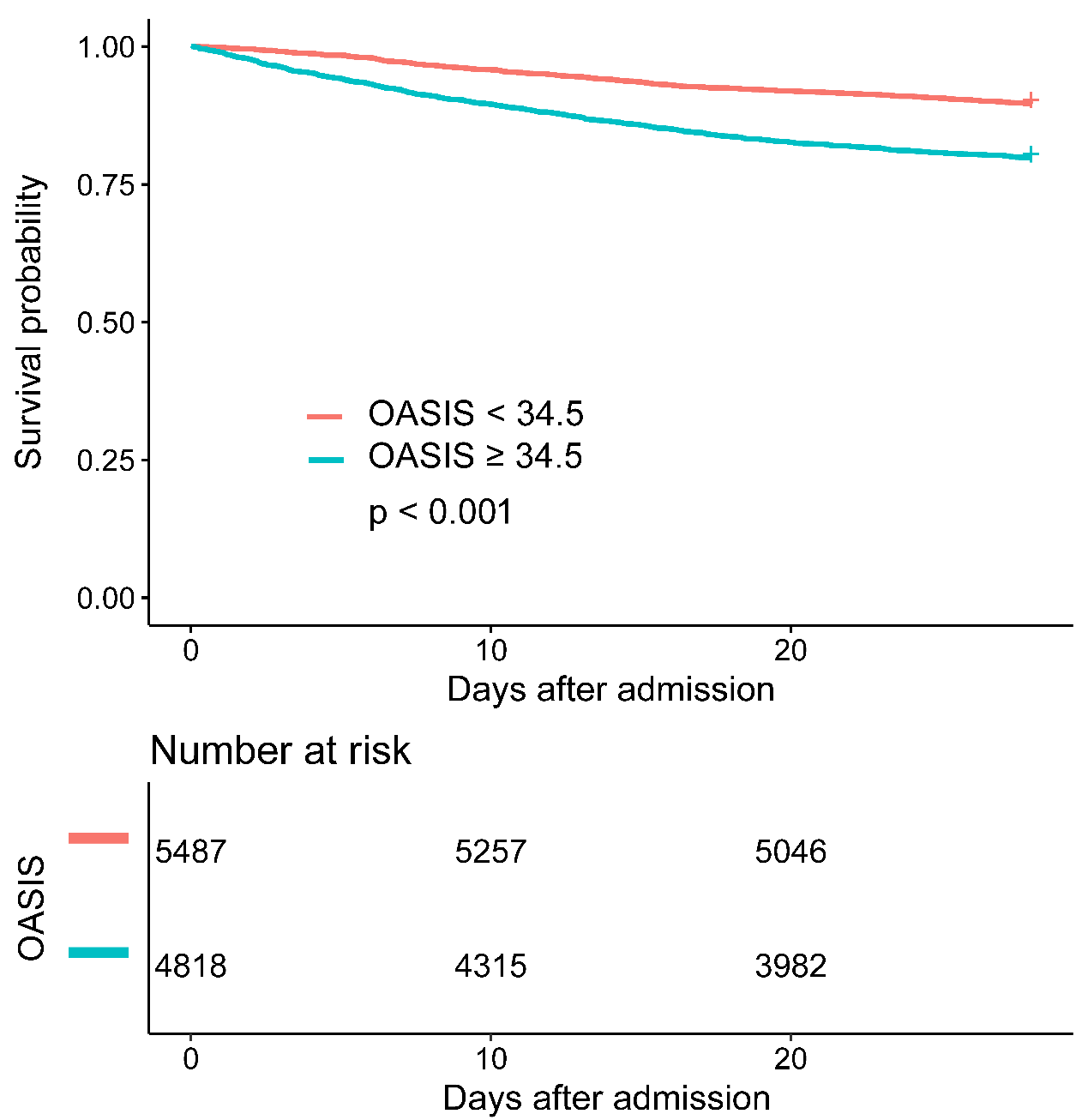

Supplement: Supplemental Information 11 — Abbreviations: OASIS, Oxford acute severity of illness score; ICU, intensive care unit. [file peerj-07-7083-s011.png]

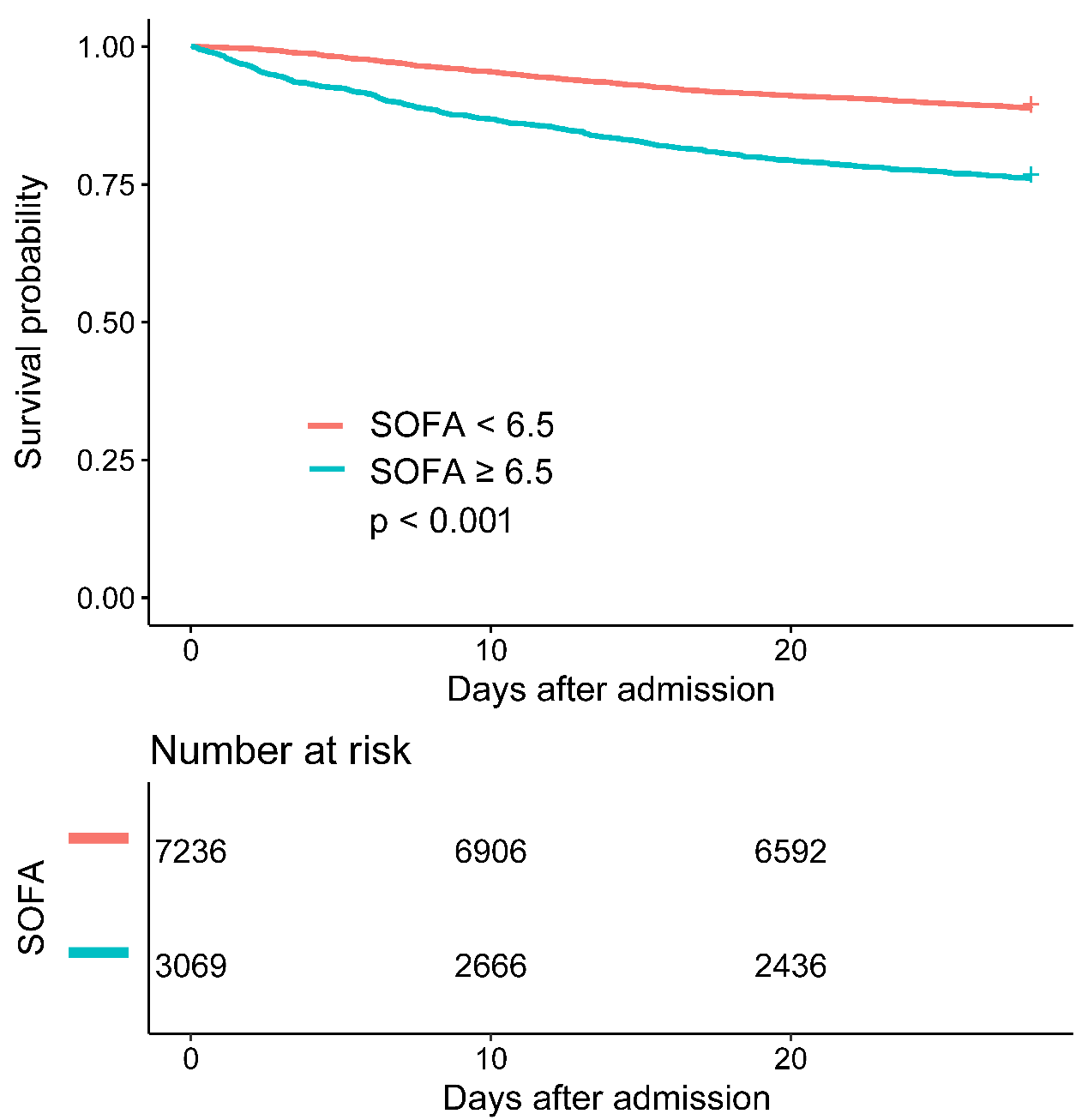

Supplement: Supplemental Information 12 — Abbreviations: SOFA, Sepsis-related organ failure assessment score; ICU, intensive care unit. [file peerj-07-7083-s012.png]

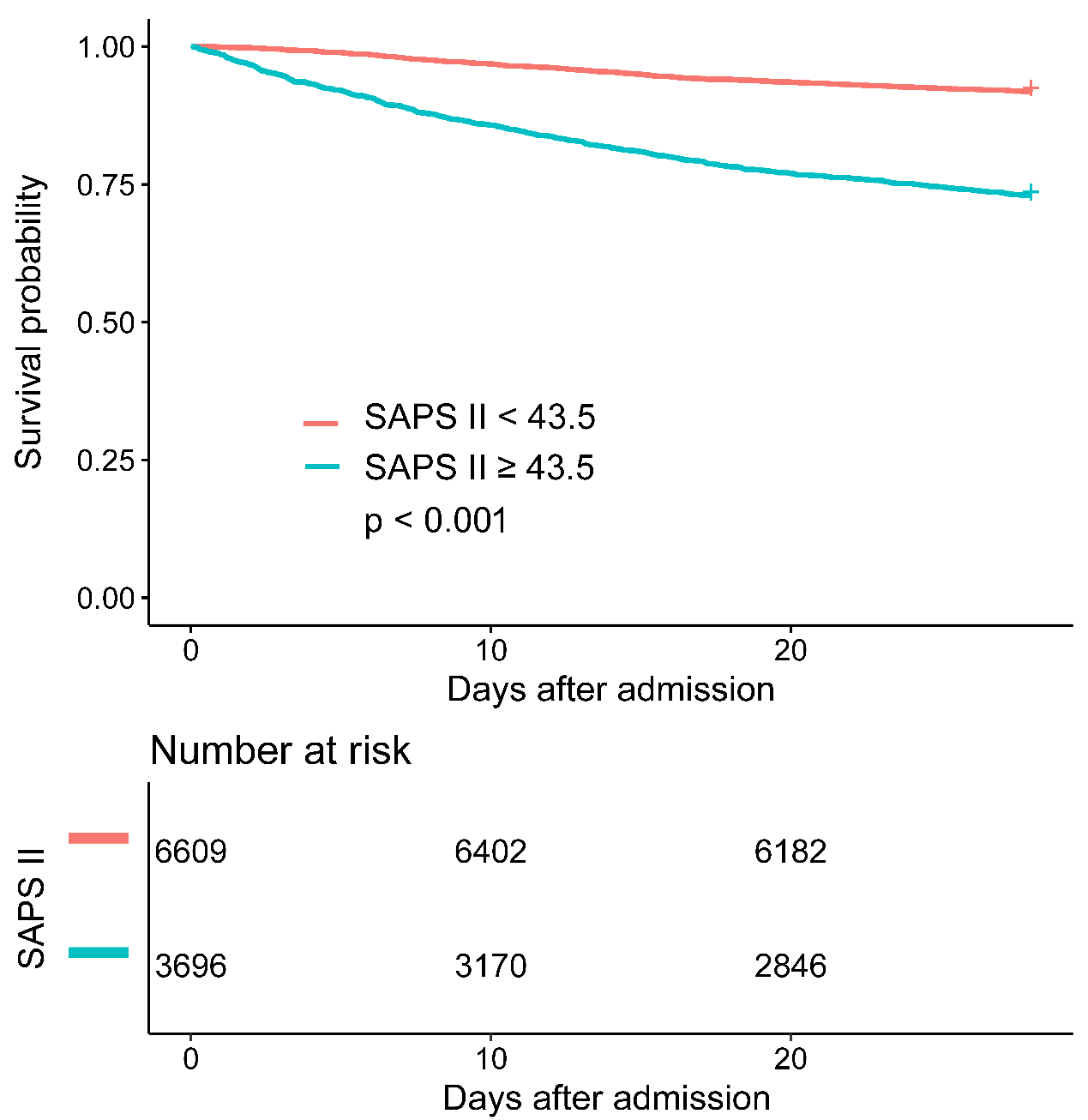

Supplement: Supplemental Information 13 — Abbreviations: SAPS II, simplified acute physiology score II; ICU, intensive care unit. [file peerj-07-7083-s013.png]
