# Supplementary material for: Prognosis predictive value of the Oxford Acute Severity of Illness Score for sepsis: a retrospective cohort study
Source: PeerJ. 2019 Jun 10;7:e7083. doi: 10.7717/peerj.7083 (PMC6563807; doi:10.7717/peerj.7083)
Supplement: Supplemental Information 14 — The main codes used for extracting data are presented. More basic codes are available at https://github.com/MIT-LCP/mimic-code. [file peerj-07-7083-s014.docx]

**Raw codes**

select d.subject_id, d.hadm_id, d.icustay_id, d.gender, d.admission_age, d.admission_type, ad.primary_diagnosis, d.ethnicity, d.los_hospital, d.hospital_expire_flag, d.los_icu, e.icustay_expire_flag, sur.survivingstatus_90d, sur.survivingdate_90d, sur.survivingstatus_28d, survivingdate_28d, e.oasis, h.sofa, spa.sapsii, o.explicit_sepsis, mcd.vent AS vent_firstday, kfc.rrt AS rrt_firstday, abscore.elixhauser_sid30, ab.congestive_heart_failure, ab.cardiac_arrhythmias, ab.valvular_disease, ab.pulmonary_circulation, ab.peripheral_vascular, ab.hypertension, ab.paralysis, ab.other_neurological, ab.chronic_pulmonary, ab.diabetes_uncomplicated, ab.diabetes_complicated, ab.hypothyroidism, ab.renal_failure, ab.liver_disease, ab.peptic_ulcer, ab.aids, ab.lymphoma, ab.metastatic_cancer, ab.solid_tumor, ab.rheumatoid_arthritis, ab.coagulopathy, ab.obesity, ab.weight_loss, ab.fluid_electrolyte, ab.blood_loss_anemia, ab.deficiency_anemias, ab.alcohol_abuse, ab.drug_abuse, ab.psychoses, ab.depression

from "icustay_detail" As d

left join "oasis" AS e on d.icustay_id=e.icustay_id

left join "sofa" AS h on d.icustay_id=h.icustay_id

left join "angus_sepsis" AS o on d.hadm_id=o.hadm_id

left join "elixhauser_ahrq_no_drg_all_icd" AS ab on d.hadm_id=ab.hadm_id

left join "elixhauser_ahrq_score" AS abscore on d.hadm_id=abscore.hadm_id

left join (select ac.hadm_id, ac.icd9_code AS primary_diagnosis from diagnoses_icd AS ac where ac.seq_num=1) AS ad on d.hadm_id=ad.hadm_id

left join "rrtfirstday" AS kfc on d.icustay_id=kfc.icustay_id

left join "ventfirstday" AS mcd on d.icustay_id=mcd.icustay_id

left join "sapsii" AS spa on d.icustay_id=spa.icustay_id

left join "surviving" AS sur on d.icustay_id=sur.icustay_id

where d.icustay_seq=1 AND d.hospstay_seq=1

AND d.admission_age>=18

AND h.sofa>=2 AND o.infection=1

AND d.los_hospital>=d.los_icu

AND d.los_icu>=1

order by d.subject_id, d.hadm_id, d.icustay_id
